# Inhalation of nebulized *Mycobacterium vaccae* can protect against allergic bronchial asthma in mice by regulating the TGF-β/Smad signal transduction pathway

**DOI:** 10.1186/s13223-020-00456-8

**Published:** 2020-07-02

**Authors:** Xiao-hong Jiang, Chao-qian Li, Guang-yi Feng, Ming-jie Luo, Qi-xiang Sun

**Affiliations:** 1 Department of Geriatric Respiratory Medicine, The First Affiliated Hospital of Guangxi Medical University, Nanning, 530021 Guangxi China; 2 The First Affiliated Hospital of Guangxi Medical University, Nanning, 530021 Guangxi China; 3 Department of Respiratory Medicine, The First Affiliated Hospital of Guangxi Medical University, Nanning, 530021 Guangxi China; 4 Department of Respiratory Medicine, Nanxishan Hospital of Guangxi Zhuang Autonomous Region, Guilin, 530021 Guangxi China; 5 The Graduate School of Guangxi Medical University, Nanning, 530021 Guangxi China

**Keywords:** Bronchial asthma, Mycobacterium vaccae, TGF-β, Signal transduction

## Abstract

**Background:**

*Mycobacterium vaccae* nebulization imparted protective effect against allergic asthma in a mouse model. The TGF-β/Smad signal transduction pathway plays an important role in allergic bronchial asthma. However, the effect of *M. vaccae* nebulization on the TGF-β/Smad signal transduction pathway in mouse models of allergic asthma remains unclear. This study investigated the preventive effect of *M. vaccae* nebulization during bronchial asthma in a mouse model and elucidate the implication of TGF-β/Smad signal transduction pathway in the process.

**Methods:**

In total, 24 female Balb/c mice were randomized to normal control (group A), asthma control (group B), and *M. vaccae* nebulization (group C) groups. Both groups B and C were sensitized using ovalbumin for establishment of the asthmatic model; group A received phosphate-buffered solution. Prior to the establishment of asthma, Group C was nebulized with *M. vaccae*. Airway responsiveness was measured in all the groups, using a noninvasive lung function machine before and 24 h after establishment of the asthmatic model. The animals were then harvested, and bronchoalveolar lavage fluid (BALF) and lung tissue were collected. The total cell counts in BALF was estimated. Protein expression of TGF-β1, TβR1, Smad1, and Smad7 was detected by immunohistochemistry. The population of CD3^**+**^γδT, IL-13^**+**^CD3^**+**^T, TGF-β^**+**^CD3^**+**^T, IL-13^**+**^CD3^**+**^γδT, and TGF-β^+^ CD3^+^ γδT cells were detected by flow cytometry. One-way analysis of variance for within-group comparisons, the least significant difference t-test or Student–Newman–Keuls test for intergroup comparisons, and the nonparametric rank sum test for analysis of airway inflammation scores were used in the study.

**Results:**

The eosinophil count; protein expression of TGF-β1, TβR1, and Smad1; and percentages of CD3^**+**^γδT and IL-13^**+**^CD3^**+**^T cells were significantly lower in the *M. vaccae* nebulization group than in the asthma control group (*P* < 0.01). There were significant intergroup differences in the percentages of TGF-β^**+**^CD3^**+**^T and IL-13^**+**^CD3^**+**^γδT cells (*P *< 0.05).

**Conclusions:**

*Mycobacterium vaccae* nebulization could confer protection against allergic bronchial asthma by reducing airway responsiveness and alleviating airway inflammation in mice. The underlying mechanism might be attributed its effect on the deregulated expression of TGF-β1, TβR1, Smad1, and Smad7 of the TGF-β/Smad signal transduction pathway.

## Background

Bronchial asthma is a serious global health concern afflicting 3 billion people worldwide, with an increasing prevalence in developed countries. The disease still imposes a severe burden on health care systems. According to the Global Initiative for Asthma (GINA) 2018 report, asthma can be controlled, but not cured. Currently, there are no effective measures to prevent the condition. Allergen-specific immunotherapy is effective for the treatment of allergic patients with symptoms. Studies on the efficacy of immunotherapy during primary and secondary prevention of asthma and allergy are at their nascent stage.

Bronchial asthma is a heterogeneous disease, characterized by chronic airway inflammation. It is often accompanied by respiratory symptoms, such as wheezing, shortness of breath, chest tightness, and cough. These vary over time and in intensity, together with the variation in expiratory airflow limitation [1]. The pathogenesis involves reversible limitation of airflow, airway hyperresponsiveness, and airway remodeling. The airway inflammation is orchestrated through multiple cell types, inflammatory mediators, and cytokines and involves a considerable number of conduction channels and regulatory networks. The manifestation of asthma is also brought about by the concerted effects of numerous pathways and large regulatory networks, such as the transforming growth factor β (TGF-β)/Smad signaling pathway [2], STAT3/NF-κB signaling pathway [3], IL-37 signaling pathway [4], Notch1-GATA3 signaling pathway [5], and many others [6–9].

The TGF-β receptor-mediated signal transduction pathway plays an important role in the pathogenesis [10], and contributes to airway inflammation, airway hyperresponsiveness, and airway remodeling of asthma. The TGF-β receptor-mediated signal transduction pathway is composed of the TGF-β superfamily, TGF-β receptor, and mothers against decapentaplegic protein (Smad) signal molecules [11].

Inactivated mycobacterium is a type of multi-functional immune regulator belonging to the Mycobacteriaceae such family as BCG (an attenuated form of *Mycobacterium bovis*). It mainly affects the immune response by regulating the immune function, enhancing T-helper (Th) cell activity, stimulating B cells to proliferate and differentiate, and eventually promoting specific antibody formation. Our former research demonstrated that inactivated mycobacteria nebulization can regulate the Th1/Th2 balance, reduce airway responsiveness, and alleviate asthmatic symptoms effectively [12, 13]. *M. vaccae* is a bi-directional immunomodulator wherein its main component is a protein of bovine mycobacterium, which is commonly used as an adjuvant therapy against tuberculosis [14–16]. Recently, it has also been used in the treatment of bronchial asthma with promising results [17, 18]. Recent studies from our group have demonstrated *M. vaccae* nebulization to have a protective effect against asthma in Balb/c mice by regulation of the Th9 cells [19].

Earlier, our research demonstrated that inactivated mycobacteria could alleviate asthmatic airway inflammation by regulating the immune system. Inactivated mycobacteria nebulization is a convenient and safe method of prevention and treatment of bronchial asthma with minimal side effects [20]. *M. vaccae* is an attenuated mycobacterium, similar to BCG and other inactivated mycobacteria. Accordingly, we presumed that the preventive and therapeutic effects of *M. vaccae* on asthma would be like those of inactivated mycobacteria.

The current study investigated the preventive effect of *M. vaccae* nebulization on bronchial asthma in a mouse model of allergic asthma and evaluated its effect on the TGF-β/Smad signal transduction pathway.

## Methods

### Main reagents and instruments

Ovalbumin (OVA) was obtained from Sigma-Aldrich (St. Louis, MO), aluminum hydroxide gel from Thermo Fisher Scientific (Waltham, MA), and *M. vaccae* injections from Anhui Chi dragon coma Biological Pharmaceutical Co. The general SP detection kit and the 3,3′-diaminobenzidine tetrahydrochloride (DAB) chromogenic reagent kit were obtained from Beijing Jinqiao Biological Technology Co., Ltd. (Beijing, China). TGF-β1 antibody, TβR1 antibody, Smad1 antibody, and Smad7 antibody were obtained from Abcam (Cambridge, United Kingdom).

Other materials and equipment used were type IV collagenase, mouse viscera lymphocyte separation solution (Haoyang, Tianjing, China), phorbol 12-myristate 13-acetate (PMA)/lonomycin mixture, fetal bovine serum, FIX&PERM Kit, PE-CY5-anti-mouse CD3, FITC-IgG1, FITC-anti-mouseγδTCR, PE-IgG1, PE-anti-mouse-IL-13, PE-anti-mouse-LAP, glycogen staining kit, Wright stain, hematoxylin–eosin (HE) staining kit, cytometer, electron microscope, high-speed cryogenic centrifuge, ultrasonic atomizer WH-2000 (Yuehua medical instrument factory, Guangdong, China), atomized inhalation box (self-made), carbon dioxide incubator, lung function machine (Buxco, USA), 10% chloral hydrate, normal saline, 10% formaldehyde, and phosphate-buffered solution (PBS).

### Ethics statement

The study was performed in accordance with the guide for the care and use of laboratory animals of the National Institutes of Health, and was approved by the Guangxi Medical University Animal Care and Use Committee (Protocol number: 20131002). All surgeries were performed under pentobarbital anesthesia and all efforts were made to minimize suffering.

### Establishment of the mouse bronchial asthma model

**Fig. 1:**
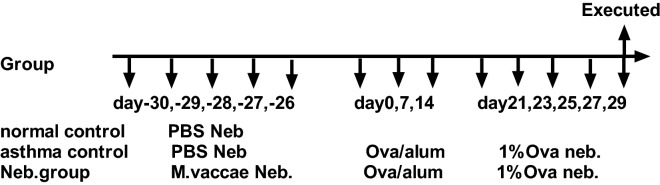
Experimental protocol. *M. vaccae*: *Mycobacterium vaccae*; Neb.: nebulization

Twenty-four 8–10-week-old pathogen-free female Balb/c mice (20–25 g) were provided by the Medical Animal Center of Guangdong Province (Guangdong, China). The mice were randomly divided into 3 groups, namely, normal group (group A), asthmatic model group (group B), and *M. vaccae* nebulization group (group C). Both groups B and C were sensitized with OVA and Group A with PBS. The mice in group C were nebulized with *M. vaccae* before the asthmatic models were established according to our former research [19]. The mice in group B were sensitized by OVA intraperitoneal injection and nebulization (overall 200 μL PBS mixed with 25 μg OVA, 1 mg aluminum hydroxide gel, and PBS liquid were injected intraperitoneally into each mouse on day 1, 8, and 15). On day 22, 24, 26, 28, and 30, the mice were nebulized with 20 mL 1% OVA fluid). OVA was replaced with PBS fluid in group A. The mice in group C were nebulized with 22.50 μg *M. vaccae* mixed with 20 mL PBS fluid once a day for 5 consecutive days (Fig. 1). Airway responsiveness was evaluated in all the mice, 24 h after the last nebulization.

### Airway responsiveness measurement

**Fig. 2:**
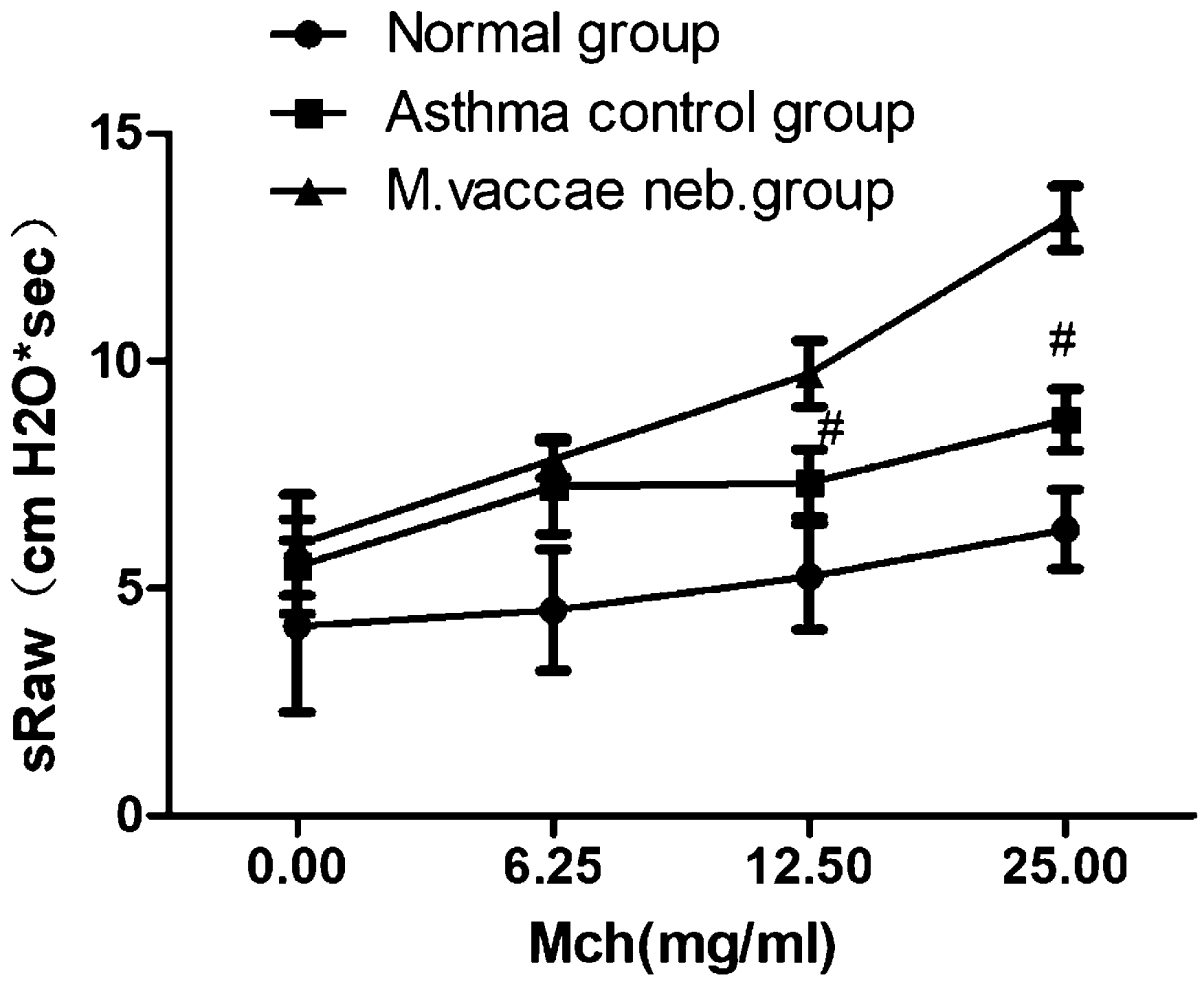
Airway responsiveness results. ^#^P < 0.01, compared with Asthma control group

Airway responsiveness was measured using a noninvasive lung function machine (Fine-Pointe™ NAM system TBL4500, Buxco, Wilmington, NC, USA) after the last stimulation with OVA. After calibration and 5 min of adaptation, the mice were nebulized with 20 μL PBS and methacholine (Mch; Sigma-Aldrich) at concentrations of 6.25, 12.5, and 25 mg/mL, respectively, for 30 s each. Data were recorded for 3 min, and the mice were allowed to recover for 4 min. The results were automatically analyzed after the experiment ended. The airway responsiveness presented as specific airway resistance (sRaw) (Fig. 2).

### Specimen collection

The mice were anesthetized using an intraperitoneal injection of 1% pentobarbital (50 mg/kg body weight), dehematized through their eyeballs, and fixed on their backs. The lungs were lavaged using 500 μL iced PBS thrice, and the bronchoalveolar lavage fluid (BALF) was collected in Eppendorf tubes on ice. The recovery of BALF was > 80%. The BALF was centrifuged to identify cytokines in the supernatant. The right upper lobes were fixed in formalin for histology and the left lobes were cut into pieces and digested with 0.1% type IV collagenase (Sigma-Aldrich) for 45 min to produce a single cell suspension. Lymphocytes in the single cell suspension were separated using mouse tissue lymphocyte separation liquid according to the operating instructions for cytometry analysis.

### Bronchoalveolar lavage fluid cell count and cell classification

The BALF was centrifuged for 10 min at 4 °C and 1500 r/min, the supernatant was discarded, and the sediment resuspended and mixed in 100 μL PBS. Of this, 30 μL was used for cell counts in the cell counting chamber, 30 μL was smeared for Wright’s staining, and 200 inflammatory cells were counted under an oil microscope. Cells were classified on the basis of morphological and staining characteristics.

### Pulmonary histopathological examination and inflammation scores

The lung tissue was fixed in 10% formaldehyde solution for 12 h. After gradient alcohol dehydration, dewaxing with xylene, and paraffin embedding, the slice was sectioned with a slice thickness of 3 µm. Both HE staining and periodic-acid Schiff (PAS) staining were performed to observe the alveolar structure, infiltration of inflammatory cells around the airway, coloration of the airway epithelium, distribution of goblet cells, and secretion of mucus. The inflammation score was evaluated based on the inflammatory cell infiltration around the airway [no infiltration (0 points); a little (1 point); more (2 points); a large number, less than a group (3 points); a large number of groups (4 points)] and presence of airway goblet cells [no (0 score); < 25% (1 score); 25% to 50% (2 scores); 50% to 75% (3 scores); > 75% (4 scores)].

### Expression of TGF-β1, TβR1, Smad1, and Smad7 protein in the lung tissue

Immunohistochemistry was used to detect TGF-β1, TβR1, Smad1, and Smad7 protein as follows: (1) preparation of lung tissue sections: the lung tissue was fixed for 12–24 h in 10% formaldehyde solution, treated with gradient alcohol dehydration, dewaxed with xylene, and embedded in paraffin. A slice of transverse section was obtained with a thickness of 3 µm. (2) immunohistochemical detection: the lung tissue slice was roasted in a thermostat at 60 °C for 3 h, then dewaxed with xylene, and washed with alcohol and water. After dewaxing, the lung tissue was repaired with citric acid solution at a high temperature and pressure for 2 min, incubated for 15 min in 3% H_2_O_2_ for blocking the endogenous peroxidase, closed for 10 min with goat serum to reduce non-specific staining, and incubated overnight in an appropriate concentration of the first antibody at 4 °C. The next morning, the slice was reheated in a 37 °C constant temperature box for 30 min and soaked completely with PBS. Then, the secondary antibody was added after removing the PBS solution.next, it was incubated with horseradish peroxidase at room temperature for 15 min and rinsed with PBS. After being incubated at room temperature for 10 min, freshly prepared DAB (20:1:1) was applied for coloring. Every sample was washed with PBS to terminate coloring, and finally the samples were colored with hematoxylin for 40 s. Next, water was dehydrated with 80, 90 and 100% alcohol. After the neutral gum was sealed, it was clustered and photographed with a microscope. Each slice was photographed under the microscope for 400 times of non-repetition. The images were analyzed using the Image-Pro Plus 6 software. The average integral light density (IOD mean) was measured.

### Estimation of the percentage of lung gamma delta T cells and detection of IL-13 and TGF-β 1 positive gamma delta T cells

(1) Single cell suspension of lung tissue preparation: the right lung was cut and crushed, then digested with type IV collagenase and filtered using a 200-mesh (aperture diameter = 0.08 mm) sieve. Lymphocytes were isolated using mouse viscera lymphocyte separation solution. (2) Lymphocyte activation: the single cell suspension of lung tissue was suspended in PBS solution. The concentration of the cells was regulated at 1 × 10^9^/L. The total amount was 300 μL. Addition of 1.2 μL PMA/Ionomycin/BFA/Monensin mixture was performed and the solution was mixed. This solution was incubated at 37 °C and 5% CO_2_ in an incubator for 4 h. (3) Estimation of the percentage of lung gamma delta T cells and IL-13, and detection of TGF- β1 positive gamma delta T cells: The specimens were divided into three tubes as A, B and C, each containing 100 μL. PE-CY5-anti-mouse CD3 10 μL was added to all the tubes. FITC-IgG1, 20 μL, was added to tube A and FITC-anti-mouse –γδTCR, 20 μL, was added to tubes B and C. These were then incubated for 15 min at room temperature. Next, 100 μL FIX & PERM Reagent A was added to the three tubes and incubated for 15 min in the dark at room temperature. Then 4 mL PBS with 5% FBS was added and centrifuged at 300×*g* and the supernatant was discarded. FIX & PERM Reagent B, 100 μL was added to all tubes. PE-IgG1, 20 μL, was added to tube A. PE-anti-mouse-IL-13, 5 μL, and PE-anti- mouse-LAP, 5 μL, were added to both tubes B and C. After vortexing for 2 s, they were incubated for 20 min at room temperature. PBS 4 mL with 5% FBS was added to each tube, and centrifuged at 300×*g*. The supernatant was discarded. Lastly, 200 μL 0.1% polyoxymethylene was added, placed at 4 °C in the dark, and analyzed using flow cytometry within 24 h.

### Statistical analysis

The data were expressed as mean ± standard deviation ($\bar{x}$ ± s) and were analyzed using the SPSS 16.0 software. One-way analysis of variance was used to analyze the comparison within groups and the least significant difference t-test or Student–Newman–Keuls test was used for comparison of quantitative data between groups. The nonparametric rank sum test was used to analyze the semi-quantitative data of the airway inflammation scores. *P* < 0.01 or *P* < 0.05 was considered statistically significant.

## Results

### Airway responsiveness

The airway responsiveness of mice was measured using an animal noninvasive lung function machine (Fig. 2).

### Bronchoalveolar lavage fluid total cell count and cell classification

**Fig. 3:**
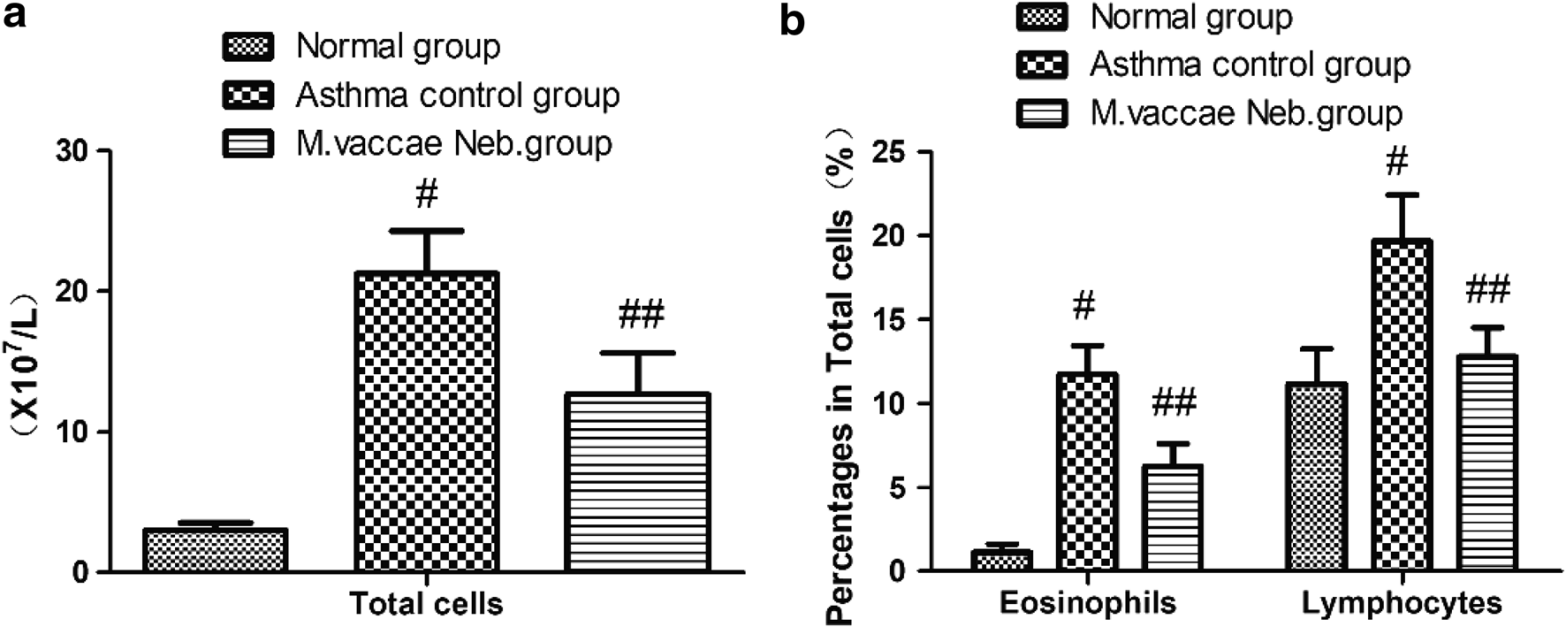
Cells count in BALF. ^#^P < 0.01, compared with Normal group; ^##^P < 0.01. compared with Asthma control group

There was a significant difference in the total number of cells, and the percentages of eosinophils, lymphocytes, and monocytes in the BALF among the 3 groups (*P* < 0.01). The total cell count and the eosinophil/lymphocyte ratio in the asthma control group were higher than that in the normal group (*P* < 0.01). The total cell count and the eosinophil/lymphocyte ratio in the nebulized group were lower than that in the asthma group (*P* < 0.01) (Table 1 and Fig. 3).

**Table 1:** Total cell count and cell classification count

Group	Total cells (×10^7^/L)	Percentages in total cells (%)			
		Eosinophils	Lymphocytes	Neutrophils	Monocytes
Normal group	3.00 ± 0.53	1.13 ± 0.52	11.19 ± 2.09	14.13 ± 1.92	73.56 ± 3.04
Asthma control group	21.25 ± 3.07^#^	11.75 ± 1.71^#^	19.69 ± 2.76^#^	16.56 ± 2.68	52.00 ± 2.89
*M. vaccae* neb. group	12.69 ± 2.94^##^	6.25 ± 1.36^##^	12.81 ± 1.73^##^	13.56 ± 1.27	67.38 ± 3.42

^#^*P* < 0.01 compared with Normal group; ^##^*P* < 0.01 compared with Asthma control group

### Pulmonary histopathological examination and inflammation score

**Fig. 4:**
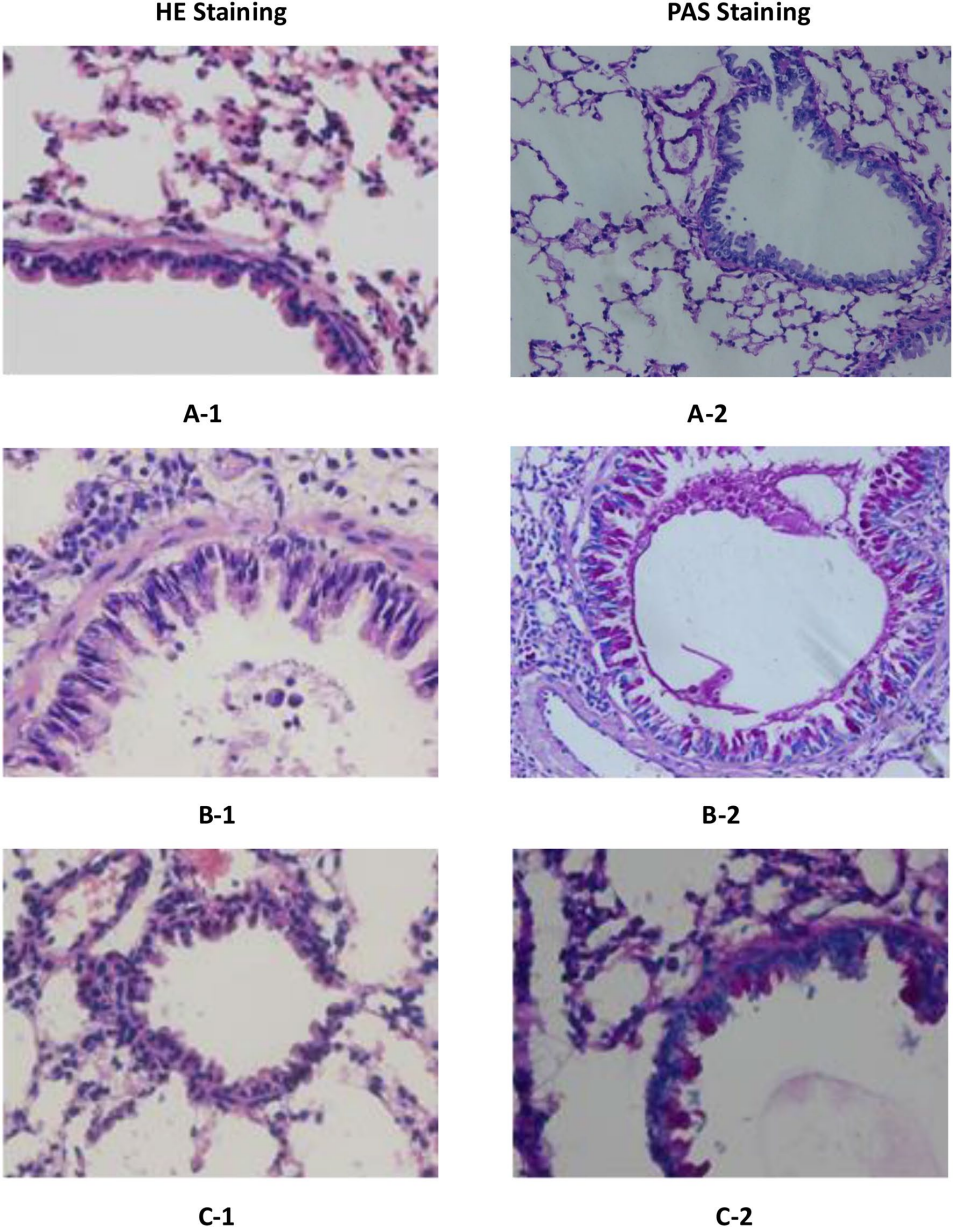
HE staining and PAS staining results of the lung tissue (×200). The lungs in the normal control group mice showed complete airway epithelium mucosa, the cilia were ordered, the basilar membranes and smooth muscle were thin, there was no inflammatory cell infiltration around the vessels and airways, there were no mucus secretion (**A1** and **A2**). In the asthma control group, there was cellular swelling of the airway epithelia, the plicae mucosae were increased, cilia were disordered, the bronchial mucous membranes were broken, many inflammatory cells infiltrated the bronchioles, the smooth muscle showed hyperplasia, PAS staining showed generous mucus and mucus plugs, (**B1** and **B2**). In the *M. vaccae* prevention group, the airway lumen was unobstructed and the epithelia lined up inorder; PAS staining showed no mucus secretion (**C1** and **C2**)

The lungs in the normal control group showed an intact airway epithelium, ordered cilia, thin basilar membranes and smooth muscle, no inflammatory cell infiltration around the vessels and airways, and no mucus secretion (Fig. 4A-1, A-2). In the asthma control group, the airway epithelial cells were swollen. The plicae mucosae were increased, the cilia were disordered, and the bronchial mucous membranes were broken. Moreover, several inflammatory cells infiltrated the bronchioles, the smooth muscle showed hyperplasia, and PAS staining showed generous mucus and mucus plugs (Fig. 4B-1, B-2). In the *M. vaccae* nebulization group, the airway lumen was unobstructed and the epithelial cells were ordered. PAS staining showed no mucus secretion (Fig. 4C-1, C-2). The semi-quantitative results of airway inflammatory cell infiltration and mucus secretion are shown in Table 2.

**Table 2:** The semi quantitative results

Group	Inflammatory cells*					Goblet cells^#^				
	0	1	2	3	4	0	1	2	3	4
Normal group	8	0	0	0	0	7	1	0	0	0
Asthma control group	0	0	1	3	4	0	0	1	2	5
*M. vaccae* neb. group	0	2	4	2	0	0	2	6	0	0

^#^*P*<0.01; *P<0.01. Kruskal–Wallis H Test

### Expression of TGF- beta/Smad signaling pathway related protein in the lung tissue

**Fig. 5:**
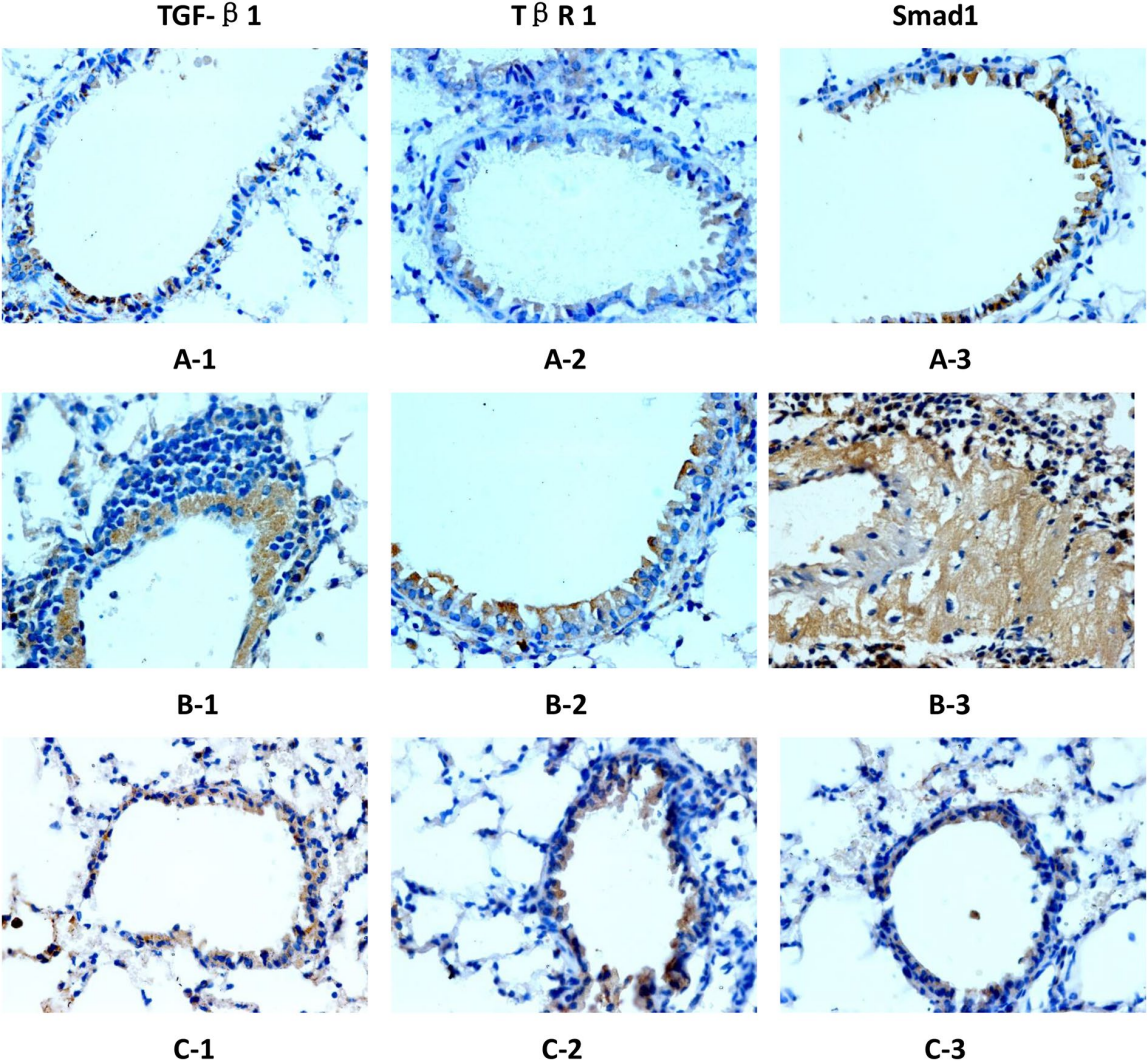
Immunohistochemistry results of pulmonary TGF-beta/Smad signaling pathway related protein expression (×400). **A1–3** (Normal control group); **B1–3** (Asthma control group); **C1–3** (*M. vaccae* neb. group)

Immunohistochemistry results showed that the proteins of the pathway were distributed in the airway epithelial cells, smooth muscle cells, macrophages, and inflammatory cells around the airway, and around the alveoli of asthmatic mice with increased inflammatory cells. The expression of TGF-β1, TβR1 and Smad1 was significantly increased in the asthma group, but was decreased in the mycobacterium nebulization group. The expression of Smad7 in the lung tissue was increased in both the asthma group and the mycobacterium nebulization group (Fig. 5).

The images were analyzed using Image-Pro Plus 6 software and the IOD mean was measured. The results were in accordance with the microscopic findings (see Table 3). Compared with the normal group, the expression of TGF-β1, T βR1, and Smad1 IOD values was significantly increased in the asthma group (*P* < 0.01), and significantly decreased in the mycobacterium nebulization group (*P* < 0.01).

**Table 3:** Pulmonary TGF-β/Smad signal transduction pathway related protein expression (IOD value)

Group	n	TGF-β1	TβR1	Smad1	Smad7
Normal group	8	8.72 ± 2.37	7.50 ± 2.50	13.60 ± 4.43	82.81 ± 28.00
Asthma control group	8	32.52 ± 15.85^#^	29.21 ± 4.73^#^	38.01 ± 18.45^#^	129.76 ± 54.20
*M. vaccae* neb. group	8	11.74 ± 4.52*	14.81 ± 4.79*	16.23 ± 7.37*	98.08 ± 35.14

^#^*P*<0.01, compared with Normal group; *P<0.01, compared with Asthma control group

### Results of flow cytometry

#### Lung gamma delta T cells and the percentages of IL-13^+^ and TGF-β^+^ in the CD3^+^ lymphocyte

Compared to the normal group, the percentages of CD3^+^ gamma delta T cells and IL-13^+^CD3^+^T cells in the lung tissue of the asthmatic group were significantly higher (*P* < 0.01). The percentages of CD3^+^ gamma delta T cells and IL-13^+^CD3^+^T cells were significantly lower in the mycobacterium nebulization group compared to the asthma group (*P* < 0.01) (Fig. 5A).

#### Lung IL-13^+^ gamma delta T^+^CD3^+^ and TGF-β1^+^ gamma delta T^+^CD3^+^ lymphocytes levels

There were significant differences in the lung IL-13^+^ gamma delta T^+^CD3^+^, TGF-β1^+^ gamma delta T^+^CD3^+^ lymphocytes among the three groups (*P* < 0.05) (Fig. 5B).

## Discussion

In this study, *M. vaccae* was used to prevent bronchial asthma in a mouse model of allergic asthma. We found that *M. vaccae* nebulization can reduce the infiltration of inflammatory cells (especially eosinophils) in the airway and contributes towards various anti-inflammatory effects.

The TGF-β/Smad signaling pathway is an important regulatory pathway that affects airway inflammation, airway hyperresponsiveness, and airway remodeling. The TGF-β superfamily is a multifunctional regulator and participates in a variety of pathophysiological processes in the airway, including alveolization, airway epithelial and endothelial barrier functions, immune cell chemotaxis, platelet aggregation, cell apoptosis, and cell differentiation and proliferation. These biological processes of the airway are implicated in the pathogenesis of bronchial asthma, and therefore, the TGF-β/Smad signaling pathway becomes an important target for asthma prevention and treatment. TGF-β is an evolutionarily conserved pleiotropic factor that regulates a myriad of biological processes including development, tissue regeneration, immune responses, and tumorigenesis. Upregulation of TGF-β ligands is observed in major pulmonary diseases, including pulmonary fibrosis, emphysema, bronchial asthma, and lung cancer. TGF-β is significantly involved in asthmatic airway inflammation [21]. Furthermore, studies have shown that TGF-β1 is correlated with basement membrane thickness in asthmatic patients [16]. Besides, TGF-β might have different expression patterns in moderate and severe asthma and the two forms of the disease might also showcase distinct variances in some of the major immunological parameters [22].

Allergic asthma is mainly manifested by Th1/Th2 immune imbalance, and this provides the basis for the use of immunomodulators in the prevention and treatment of asthma. Our previous study demonstrated that *M. phlei* inhalation plays an anti-inflammatory role in the prevention and treatment of asthma. This research is aimed at combining these two intervention points to provide new ideas and a theoretical basis for the prevention and treatment of asthma.

*Mycobacterium vaccae* has been used in the treatment of tuberculosis as an immunomodulator. Researchers have found that intranasal administration of recombinant *M. smegmatis* can attenuate airway inflammation in a murine model of allergic asthma [23]. Furthermore, heat shock protein X, (HspX) purified from *M. tuberculosis,* plays a critical role in the amelioration of asthmatic inflammation in mice [24].

Eosinophil chemotaxis is an important pathological feature of asthma. It causes release of toxic protein particles and reactive oxygen free radicals, and secretion of cytokines and lipid mediators [25]. In addition, eosinophils are an important source of TGF-β, and they have a linear relationship. Thus, we surmise that *M. vaccae* nebulization can protect against bronchial asthma in mice by regulating the immune function of the airway and reducing eosinophil infiltration, which may also be an important mechanism for the regulation of the TGF-β/Smad signaling pathway. This study also found that *M. vaccae* nebulization could reduce the eosinophil ratio in asthmatic mice, which was dependent on the expression of TGF-β1, TβR1, and Smad1 protein of the TGF-β/Smad signaling pathway. However, it had little effect on the negative regulation of Smad7 protein in this pathway. The TGF-β family participates in airway remodeling in asthma. Its major role, in asthma, is to promote chronic inflammation, promote the secretion of inflammatory mediators, and induce chemotaxis, aggregation, and infiltration of inflammatory cells in the asthmatic airway. It promotes the differentiation and proliferation of airway epithelial cells, fibrosis of epithelial tissue, proliferation of smooth muscle, and airway epithelial-mesenchymal transition. Chiefly, TGF-βa acts through the TGF-β/Smad signaling pathway, wherein the TGF-β ligand family combines with the TβR I and II receptors on the surface of the target cells and activates the intracellular Smad1 protein to form the Smad protein complex. Play the cell signal into the nucleus and plays a role in the cell nucleus. However, *M. vaccae* nebulization cannot block the signal transduction pathway completely, which has both positive and negative regulation. The overall effect seems to be one of positive regulation.

## Limitations

This study is limited by the fact that the regulatory effects of *M. vaccae* were evaluated only in small animals. Further clinical studies are warranted to validate our findings.

## Conclusions

We infer that *M. vaccae* nebulization can protect against bronchial asthma in mice by regulating the immune function of the airway and reducing eosinophil infiltration, which may also be an important mechanism for the regulation of the TGF-β/Smad signaling pathway. Thus, the study provides new ideas for the prevention and control of bronchial asthma by using the immunomodulatory and TGF-beta/Smad signaling pathways as targets.

## Data Availability

Data may be shared but not copied due to related ongoing research projects in our lab.

## Abbreviations

BALF: Bronchoalveolar lavage fluid
DAB: 3,3′-Diaminobenzidine tetrahydrochloride
HE: Hematoxylin-eosin
GINA: Global initiative for asthma
IOD mean: Average integral light density
OVA: Ovalbumin
PAS: Periodic-acid Schiff
PMA: Phorbol 12-myristate 13-acetate
TGF-β: Transforming growth factor β
